# Prognostic modeling of predictive maintenance with survival analysis for mobile work equipment

**DOI:** 10.1038/s41598-022-12572-z

**Published:** 2022-05-20

**Authors:** Zhen Yang, Juho Kanniainen, Tomi Krogerus, Frank Emmert-Streib

**Affiliations:** 1 Faculty of Information Technology and Communication Sciences, Computing Sciences, Tampere University, Korkeakoulunkatu 10, Tampere, 33720 Finland; 2 Cargotec Oyj, Ruskontie 55, Tampere, 33710 Finland; 3 Predictive Society and Data Analytics Lab, Faculty of Information Technology and Communication Sciences, Tampere University, Korkeakoulunkatu 10, Tampere, 33720 Finland

**Keywords:** Computational science, Statistics, Engineering

## Abstract

In recent years there is a data surge of industrial and business data. This posses opportunities and challenges at the same time because the wealth of information is usually buried in complex and frequently disconnected data sets. Predictive maintenance utilizes such data for developing prognostic and diagnostic models that allow the optimization of the life cycle of machine components. In this paper, we address the modeling of the prognostics of machine components from mobile work equipment. Specifically, we are estimating survival curves and hazard rates using parametric and non-parametric models to characterize time dependent failure probabilities of machine components. As a result, we find the presence of different types of censoring masking the presence of different populations that can cause severe problems for statistical estimators and the interpretations of results. Furthermore, we show that the obtained hazard functions for different machine components are complex and versatile and are best modeled via non-parametric estimators. However, notable exceptions for individual machine components can be found amenable for a Generalized-gamma and Weibull model.

## Introduction

Machine prognostics and diagnostics have received much attention in recent years^1–3^. Its ultimate goal is to derive preventive maintenance schedules for reducing machine downtime and maintenance costs and for improving spare part supply chains to optimize warehouse organization^4–7^. Hence, comprehensive machine prognostic and diagnostic systems are anticipated to provide enormous benefits for organizations operating service or production machinery.

One major challenge for machine prognostics is the modeling of the reliability of machine parts or its components. For this purpose, machine failure data have been utilized for modeling the reliability of machine components in combination with reliability theory. Interestingly, reliability theory and survival analysis^8^ have essentially the same definitions but survival analysis has a longer history with many applications in the biomedical sciences^9,10^. For this reason survival analysis has been identified as having great potential of becoming a major tool for modeling machine reliability^11^. In this paper, we contribute an analysis investigating the parametric and non-parametric modeling of the reliability of machine parts by using a number of different methods from survival analysis.

Survival analysis was initially introduced for the analysis of life tables^12^. However, the meaning of an ’event’, upon which a survival analysis is based on, is not limited, e.g., to the death of biological organisms or humans but it is far more general and can also correspond to graduation of a student, crime or divorce^13–15^. Hence, the application of survival analysis is very flexible, and this is the reason for its widespread usage. Furthermore, this flexibility explains also the existence of multiple synonyms used instead of survival analysis by different fields, e.g., event history analysis, duration analysis or reliability analysis^16^. A major problem in studying survival data, also known as time-to-event data, is the presence of censoring^17,18^. In general, censoring refers to a form of missing data in which the time-to-event is for some reason not observed. Interestingly, in contrast to other types of missing data, e.g., due to sensor failure or the accidental deletion of information^19^, censoring is not due to any error or mistake. Instead, it represents an unavoidable characteristic of the phenomenon under investigation. For instance, in a clinical study patients may decide to participate no longer in a clinical trial and to drop out before the event of interest occurs. Similarly, the customer of a service plan for a production machine may decide to discontinue a service contract before the failure of a component. In order to deal appropriately with censoring, dedicated statistical analysis methodology has been developed^20^. Importantly, such methods require the careful selection because the application of inappropriate methodology leads to systematic errors in an analysis and renders its interpretations as meaningless.

In the context of machine prognostics, so far only a limited number of studies have been conducted using survival analysis in the presence of censoring for investigating machine component failures^21^. For instance, from a Google Scholar we find only 568 publications in the context of predictive maintenance while for clinical applications over 1.5 million publications can be found. An early example is from^22^ where the authors proposed a three-parameter Weibull distribution to model the time to failure data from overhead line components subject to right-censoring. In^23^, the authors applied survival analysis technique for the modeling of the failure probability of replaced joint. In their paper, they discussed issues regarding employing survival analysis for drawing survival probability on highly censored-data which sometimes is the common issues for machine failure data. The paper by^24^ focuses on supporting and improving the preventive identifying and replacement on the water pipe using survival analysis technique, the author analyzed the hazard rate between groups and investigated the hazard ratio for different risk factors by non-parametric and semi-parametric methods. General reviews about different machine learning aspects of predictive maintenance and machine diagnosis can be found in^25–27^.

In addition to the above, there are also more distance studies using survival analysis. For instance, in^28^ a simulation study was presented comparing Kaplan Meier Estimator (KME), the Piecewise Exponential Estimator (PEXE) and the Maximum Likelihood Estimator (MLE) for estimating survivor functions. Yet a different type of study aims at combining survival analysis with machine learning approaches. For instance, in^29^, the authors discussed several machine learning related approaches in modeling the remaining useful life (RUL) of industrial robots. In their paper they used COX Proportional Hazard Model (CPHM) for modeling RUL, and they make comparisons between a CPHM, extremely randomized trees, K-nearest and convolutional neural network for the estimation of the RUL of robots. Also in^30^ survival analysis and machine learning were combined to form a probability-based support vector machine to predict remaining life of bearings components. Finally, we would like to mention that there are reviews of reliability modeling which include also some discussions about survival analysis, see, e.g.,^11,21^.

The main purpose of this paper is to study the modeling of the prognostics of machine components from mobile work equipment. Specifically, we are estimating survival functions and hazard functions using parametric and non-parametric models to characterize time dependent failure probabilities. As a result, we find the presence of different types of censoring that can cause severe problems for the statistical estimators. Furthermore, we show that the obtained hazard functions for different machine components are complex and versatile and are best modeled via non-parametric estimators. The methods and results of this paper can be used to develop an actual expert system for predictive maintenance with realistic hazard functions within various environments.

In contrast to other papers, our study makes the following contributions. First, while survival analysis has been used before in studies about predictive maintenance, usually, parametric models are used^22,31,32^. However, the assumptions that need to hold for parametric models to be applied are rarely checked. For this reason, we compare in this paper parametric and non-parametric models to reveal the influence of parametric assumptions. Second, survival analysis is an advanced statistical method that needs to be applied with care. In this respect, not only the method is of importance but also the quality of the data. Since time-to-event data are not directly measurable one needs to reconstruct these from the available information. In our case the distinction of the number of maintenance events is key for obtaining well-defined data allowing a sound analysis. Furthermore, also distinguishing between different types of censoring is important because otherwise the statistical methodology for estimators suffers. Third, the data we use in our study are real-word data from from cargo and load handling equipment. The data have been acquired from Cargotec and represents a unique and complex data set for which no reference exists so far in the literature. Hence, our analysis enters unchartered territory for this research domain. Forth, our analysis does not stop with the estimation of hazard rates but includes a categorization of the studied machine components. This allows to identify different classes of characteristic behavior which could be exploited for a downstream analysis, e.g., for a more efficient organization of supply chains.

We organize the paper as follows. In Section “Methods” we discussed the underlying methodology of our work, as well as a brief introduction to our data. In Section “Data pre-processing” we discuss our efforts on identifying and removing possible noises from our data, also how we form and format our input data. In Section “Survival curves for individual components” we employ non-parametric estimation to demonstrate the survival probability from 12 specially selected components from a specific type of mobile work equipment. In Section “Non-parametric and parametric estimation of hazard rates” we shows the hazard estimations for 12 selected components using the non-parametric method and 5 parametric methods. In Section “Assessing the parametric models of the hazard rates” the numerical goodness of fit metrics from 5 parametric models are listed. Section "Discussion" we compare the hazard curves from different parametric models on different parts graphically and numerically by accessing the goodness of fit using the presented metrics, after which we discuss the feasibility of applying parametric models, also we compare our work to some other similar works. In the Conclusion we summary main findings and the contributions of our work.

## Methods

In this section, we present all methods and data we use for our analysis. Furthermore, we clarify the meaning of terminology.

### Methods from survival analysis

For conducting a survival analysis of machine components, we need to estimate a survival function and a hazard function^33^. Furthermore, for the hazard function, we discuss parametric and non-parametric estimators.

#### Survival function

Simply, a survival function describes the probability of a population surviving past a certain time. Formally, a survival function is indicated by *S*(*t*) which is defined by

1 $$\begin{aligned} S(t) = \Pr ({T > t}), \end{aligned}$$

where $T>0$ is a random variable denoting the time of the failure of a component. According to the definition of a cumulative distribution function, a variable *T* smaller or equal to *t* can be written as

2 $$\begin{aligned} F(t) = \Pr ({T \le t}). \end{aligned}$$

From this follows that

3 $$\begin{aligned} S(t) = 1 - F(t). \end{aligned}$$

Since *S*(*t*) is a probability, there exists a probability density function *f* with

4 $$\begin{aligned} S(t) = \int _{t}^{\infty } f(u)du. \end{aligned}$$

#### Hazard function

In addition to the survival function, *S*, and its underlying density, *f*, there is a hazard function, *h*. Formally, the hazard function is defined as

5 $$\begin{aligned} h(t) = \lim _{\Delta t \rightarrow 0} \frac{\Pr (t\le T<t+\Delta t | T\ge t )}{\Delta t}. \end{aligned}$$

The hazard function has the interpretation of an instant probability because $\Delta t$ goes to zero.

From this one can derive an important relation between the three entities *S*, *f* and *h* given by^33^

6 $$\begin{aligned} h(t) = \frac{f(t)}{S(t)}. \end{aligned}$$

In contrast to survival function that measures the probability of surviving, the hazard function measures the instant probability that an individual will fail to survive in the time interval $(t,t+\Delta t)$ given that the individual has survived until time *t*. We would like to note that the hazard function is frequently called instant probability^34^ because it assesses the hazard rate for $\Delta t$ approaching zero (see Eq 5).

In most cases, we are more interested in the hazard function than the survival function in a survival analysis because it provides information about the failure probability.

Sometimes it is useful to know the cumulative hazard of a population accumulated over time. For this one can either take the integration over the hazard if explicitly given or one can use the Nelson-Aalen estimator^35,36^ to estimate it directly from the data. The Nelson-Aalen estimator has the form

7 $$\begin{aligned} H(t) = \sum _{t_{i}\le t}\frac{d_{i}}{n_{i}}. \end{aligned}$$

The Nelson-Aalen estimator is a non-parametric estimator that directly estimates the cumulative hazard from the data. Here $d_{i}$ is the number of events at time $t_{i}$ and $n_{i}$ is the number of individuals left at time $t_{i}$.

### Non-parametric and parametric estimators

In general, non-parametric methods do not require the data to follow any distributions as they provide a direct estimation from the data itself. Hence they have an advantage for data that do not correspond to any standard distributional form^37^. In the following, we discuss a commonly used non-parametric method for the survival function and the hazard function but also some parametric models for the hazard function.

#### Non-parametric estimation of the survival function

For estimating survival functions, we use the Kaplan-Meier estimator. The Kaplan-Meier estimator was proposed in 1958^38^. It is an non-parametric estimator for measuring the fraction of living subjects after a certain amount of time regarding one or more specific events (treatment, failure). Its major advantage in analyzing survival data is its ability to account for censored data. Due to its simplicity and its ability of modeling incomplete observations, it is one of the best non-parametric methods to estimate the survival^39^ for data.

Formally, it is given by

8 $$\begin{aligned} S(t) = \prod _{i:t_{i}<t} \left( 1-\frac{d_{i}}{n_{i}} \right) . \end{aligned}$$

Here $n_{i}$ is the number of subjects living at time step *i*, and $d_{i}$ denotes the number of events that happened at time *i*. The inputs to the Kaplan-Meier estimator are the individuals’ event time in ascending orders, Kaplan-Meier estimator recalculates the survival probability whenever a new uncensored event occurs at next time step. Kaplan-Meier survival curve is given as the probabilities of surviving regarding different time intervals, each interval is defined by the time between two successive uncensored events, consequently Kaplan-Meier curve is a step curve where the survival probability between two uncensored events remain unchanged though the censored events can continue to happen inside each interval. The Kaplan-Meier survival curve starts from 1 when all subjects are alive and decreases to 0 when all subjects experience events.

#### Non-parametric estimation of the hazard function

Non-parametric estimation of hazard has been applied widely for survival analysis, as it makes no assumptions on the data and presents an intuitive understanding of the instant failure rate.

Non-parametric estimators for the hazard rate can be mainly divided into two categories: histogram-based estimator and kernel-based smoother estimator. While both of these estimators are capable of estimating the hazard from data, the kernel method results in less biased estimations^40^. For the past ten years, kernel based smooth estimators have been widely studied and applied^41^. Therefore, in our work, non-parametric hazard was estimated using the kernel-based smoother estimator.

Non-parametric hazard estimation often involves smoothing technique that is applied on the initial estimation of the hazard since the original hazard estimation is often with high variances, a proper smoothing technique provides a good trade off between the bias and the variance hence it can improve the statistical performance of the naive hazard estimation^42^. For a set of data that contains random censorship, the kernel non-parametric estimation of hazard often can be done by smoothing the increments of the Nelson-Aalen cumulative hazard estimator using a kernel function, the kernel smoothing hazard estimation has the basic form of

9 $$\begin{aligned} h(t) = \int \left( \frac{1}{b} \right) K \left( \frac{t-x}{b} \right) dH_{NA} (x), \end{aligned}$$

where *h*(*t*) is the kernel hazard estimation, $H_{NA}$ is the Nelson-Aalen cumulative hazard estimation, *b* is the bandwidth which is a critical parameter for balancing the bias and variance by changing the degree of smoothness of the curve, usually, a bad choice of the bandwidth results in a under-fitting or over-fitting curve. $\int \left( \frac{1}{b} \right) K \left( \frac{t-x}{b} \right)$ is the kernel smoother and *K* is the kernel function. Many research works have shown that the choice of different kernels has a minor effect on the final estimation, therefore Epanechnikov kernel is often adopted in most of the studies^41^, and its kernel function can be written as:

10 $$\begin{aligned} K(x) = \frac{3}{4}\left( 1 - x^{2}\right) \; \text{ for } |x| \le 1. \end{aligned}$$

In general, the kernel estimator can be seen as a convolution of the Nelson–Aalen estimator with a kernel function. Due to the nature of kernel functions, estimations near the boundaries are typically highly biased. Hence, corrections need to be applied. Details for the choose of the parameters for the kernel hazard of our work will be discussed in Section "Discussion".

#### Parametric models for the hazard function

Parametric models can be generalized to all types of survival models in which their distribution of the survival times are specified by a set of parameters $\theta$. Parametric survival models can be divided into fully parametric and semi-parametric types. While the fully parametric models make strong assumption on the distribution of the survival time whereas semi-parametric models do not require such assumptions. In this paper we will apply and discuss fully parametric models.

The procedure for fitting a fully parametric survival models can described as follows: Firstly estimate the non-parametric hazard rate from the data, and make proper assumptions on the shape of the curve. Then compare of the assumed shape with the hazard shapes from various parametric distributions and find a parametric distribution that fits best to the hazard shape^43^. Parametric models are widely used by many studies^44^ when the underlying distribution of data is known, they will allow a convenient representation of complex data structure^45^.

Parametric models are characterized by a set of parameter $\theta$, the form of the probability density function, cumulative probability and survival function can be rewritten as $f(t;\theta )$, $F(t;\theta )$, $S(t;\theta )$ respectively. Some of the most commonly used parametric models for survival analysis are the Exponential, Weibull, Log-logistic, and Log-normal^46^ model. In addition to those four models we also include the Generalized-gamma model which has more varieties in its hazard shape. For our analysis, the parameters from the parametric models are estimated by maximizing the full log-likelihood with respect to the parameter $\theta$.

**Figure 1:**
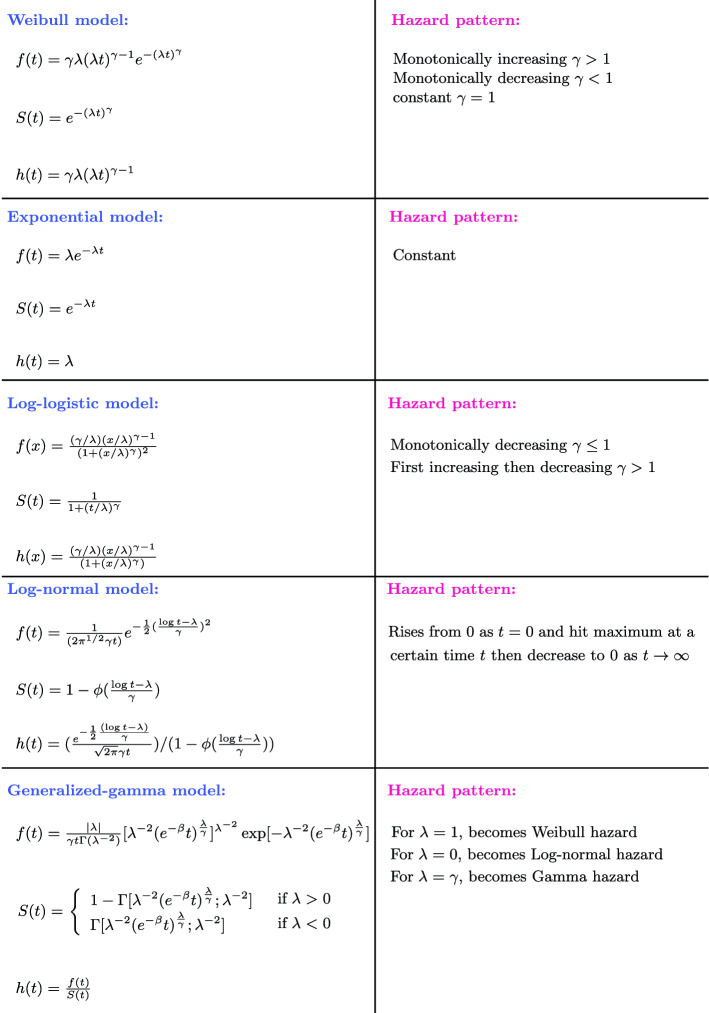
Overview of five parametric models used for our analysis. The left-hand-side provides information about the functional form of the survival density *f*, survival function *S* and the hazard rate *h* and the right-hand-side summarizes the resulting hazard-shape of the corresponding models.

Figure 1 shows the properties of those 5 parametric models including their behaviour on the shape of the hazard, their probability density, survival function, and hazard function. This gives a general overview of these parametric models and highlights also the types of behavior that can be accomplished with the corresponding hazard function.

### Model selection of parametric models

For the evaluation of the goodness-of-fit of parametric models, we use two different metrics, the Akaike information criterion (AIC) and log likelihood value (LLV).

The Akaike information criterion (AIC)^47^ is a general score used for evaluating the model fit. It is widely used to compare the goodness-of-fit between different models. Formally, it is defined as

11 $$\begin{aligned} AIC = -2l + 2(p + k), \end{aligned}$$

where *l* is the log-likelihood of the fitted model, *p* is the number of total covariates in the model, and *k* is the number of parameters in the model. AIC is advantaged when comparing models with different numbers of parameters since in the formula it takes into account of the number of the parameters from the model as a penalty to the AIC score. In general, the smaller the AIC score the better is the model fit.

Log likelihood value (LLV) is simply the log transform of the likelihood from the maximum likelihood estimation. The larger the LLV is the better the fitted model and the value of the LLV can range from −Inf to +Inf.

### Time-to-event data

For our analysis, we use time-to-event data from mobile work equipment. Specifically, we are collecting maintenance records from a specific type of mobile work equipment provided by Cargotec.

#### Censoring of data

Censoring is a characteristic of real-life data referring to the inability to observe the occurrence of completed events for individuals. In a clinical context, this can happen due to the loss track of the patient or the loss of occurrence time of event, for examples, a patient can drop out in the middle of a study or the event of interest does not happen to the patients before the end of the study period, such individuals are so-called censored individuals in survival analysis. Censorship basically exists in every time-to-event dataset and censorship is the key concept that distinguishes survival data from other types of data^17^.

**Figure 2:**
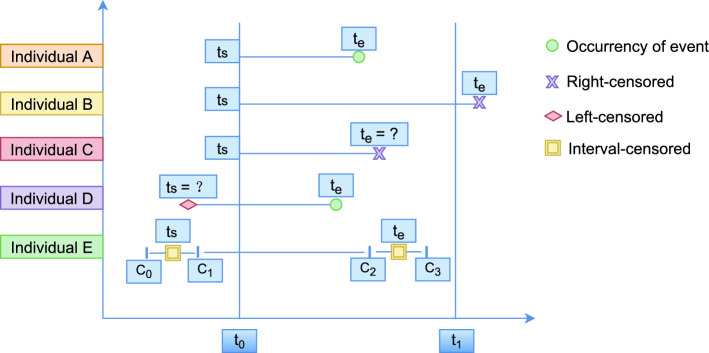
Illustrations of different censoring types. Individual A is a complete record, individual B and C are right-censored, individual D is left-censored while individual E is both left- and right-interval censored.

Left censoring, right censoring and interval censoring are three main types of censorship we can encounter in survival data. Let’s assume $t_{s}$ is the start time that an individual has been under the risk of the interested event, $t_{e}$ as the occurrence time of the interested event, $t_{0}$ as the starting time of the study, and $t_{1}$ as the ending time of the study, an illustration of different censoring types can be seen in the Fig. 2.

As all the subjects entered the study scope at different time stamp, we will scale their entry times to be $t_{0}$ for whose entry times are $\ge t_{0}$. An individual has an uncensored event when $t_{e}$ is observed during the study and $t_{s} = t_{0}, t_{e} \le t_{1}$. In Fig. 2, individual A is uncensored (meaning it has a complete observation). An individual was said to be right-censored observation when $t_{s} = t_{0}$ but $t_{e} > t_{1}$ or $t_{e}$ is unknown. In Fig. 2, individual B and C are typical types of right-censored data. Left-censoring refers to the cases that we observe $t_{e}$, $t_{e} \le t_{1}$ but the patient has been in the risk of the interested event before enrolling into the study, specifically $t_{s} < t_{0}$ and $t_{s}$ is not known. In Fig. 2, individual D is left-censored data. Interval censoring arises when we can only observe $t_{s}$ or $t_{e}$ up to a time interval, which can be seen from the individual E in Fig. 2, where $t_{s}$ is only observed to lie within the time interval $c_{0}$ and $c_{1}$, or $t_{e}$ is observed to happen in between time $c_{2}$ and $c_{3}$, these two cases can be referred as left interval censoring and right interval censoring respectively, both left and right interval censoring could happen at the same time for an individual.

In this paper, we need to deal with left-, interval- and right-censored because our data can contain all of these types of censoring. However, for our analysis, we will only keep the right-censorship while we try to identify and remove as many left- and interval-censored data as possible. Details will be presented in Section “Data pre-processing”. Also it is important that we need to specify the right-censoring types existing in the data, different types of right-censoring records sometimes could be the result of different risk factors hence have different underlying empirical distribution, therefore the type of right-censoring needs to be addressed during the analysis of survival data^23^. Therefore, the remaining right-censored data will be all of individual-B type from Fig. 2, as we assume that all the right-censored maintenance records are from the components that have been running from the installation date till now without any failures.

#### Data from cargo and load handling equipment

The type of mobile work equipment we analysis in our paper contains thousands of components and it is usually used for the transportation of goods, therefore, the main workload of the mobile work equipment can be characterized by drive distance and fuel consumption and lifted weight. We have collected maintenance records on thousands of different components for this specific type of mobile work equipment from Cargotec. Each record corresponds to one replacement event for a specific component from one machine. The 5 main columns from the maintenance records are:

- *Machine ID* unique identifier of each machine
- *Maintained part* for each maintenance event we replace the broken part with a new one.
- *Start date* the installation date of the machine if the part was maintained for the first time, else it should be the maintenance date from previous maintenance record
- *Maintenance date* date of current maintenance event.
- *Maintenance period* the period in days between the start date and maintenance date.

In order to utilize survival analysis, we assume that the installation date is the start date, and the maintenance date the event date. Whenever a component fails, such a component is replaced. Furthermore, there can be multiple maintenance events for the same component from one machine. For the records of the maintained components, their start date corresponds to the date of the last maintenance and the end date corresponds to the date of the next maintenance, however, due to the fact that those records could potentially be left-censored, in our analysis the uncensored records will only contain records of first time maintenance. A detailed discussion of this is provided in Section “Data pre-processing”.

The maintenance records we use are from thousands of different parts and components from mobile work equipment. In order to perform an analysis for representative and important components we used expert knowledge to reduce the number of components for which we provide an analysis. Specifically, we selected 12 independent components that are not sub-parts of some complex components and are among the most frequently replaced parts.

**Figure 3:**
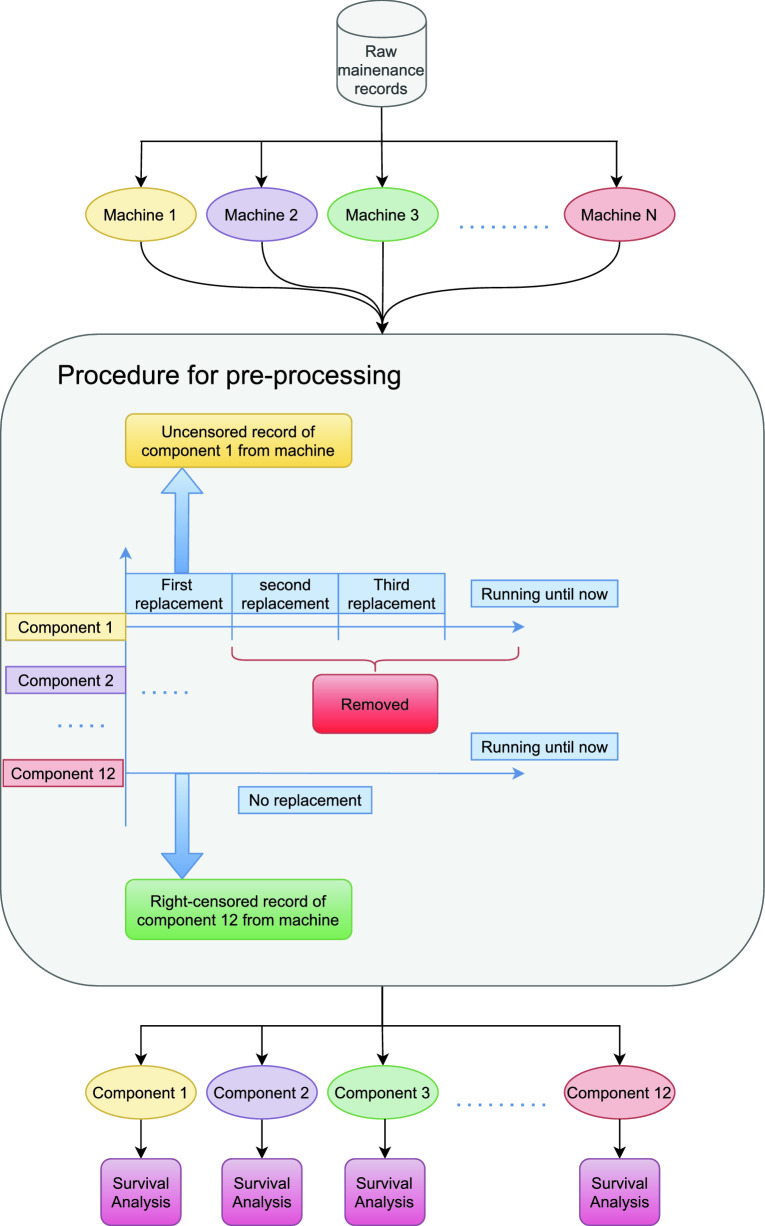
Data pre-processing diagram. This diagram shows how we obtained our survival data from the raw maintenance records.

Figure 3 summarizes all of our pre-processing steps. The raw records are grouped from different machines, then the procedure of pre-processing is applied to the records from the selected 12 components from each machine individually. During the pre-processing, if there are multiple maintenance events for a component, then the first maintenance event was kept and the others are removed. If one component from a certain machine has not been recorded for any maintenance events, then we will record this as one right-censored record.

### Definitions of important entities

The time-to-event data which we use for our analysis have a well-defined meaning. In the following, we would like to highlight some terminology we use throughout the paper in order to clarify their meaning. In general, an event indicates a certain point in time. Usually, events are commonly used as a starting point. In our case such starting points refer to the initiation of a mobile work equipment. It is clear that different mobile work equipments are initiated at different times. For this reason the information provided by an event is relative to the time of initiation. Furthermore, due to the fact that components of a mobile work equipment can fail multiple times, we need to distinguish between different types of events. Specifically, the event of the first replacement of a component is called the $1{\rm st}$ maintenance. Similarly, we call the second or $n{\rm th}$ event the $2{\rm nd}$ maintenance or $n{\rm th}$ maintenance respectively. If we want to refer to all events after but including the $n{\rm th}$ maintenance we will call it $n{\rm th}{\tiny +}$ maintenance. For instance, events with $2{\rm nd}+$ maintenance refer to all the events of the components that are replaced for the second time or more often.

## Results

In this section, we present results of our analysis. First, we present details for the preprocessing of the data. This is important because dealing appropriately with different types of censoring events is key for a sound survival analysis. Second, we show results for survival curves and hazard rates estimated from parametric and non-parametric models. Third, we present results for the goodness-of-fit of the parametric models.

### Data pre-processing

In order to apply survival analysis, time-to-event data is needed with well-defined events. This includes the appropriate identification of censoring events. Inaccurate information thereof will lead to misleading analysis result and defect its meaning. For this reason, in the following sections, we will discuss details of pre-processing steps needed for preparing the raw data.

#### Left- and interval-censored records

A general problem when using time-to-event data is the type of censoring. Basically, there are three main censoring types: Right-censoring, left-censoring and interval-censoring. This is important to note because depending on the type of the censoring, statistical estimators need to be selected whereas the most developed and best tested estimators are for right-censoring data.

**Figure 4:**
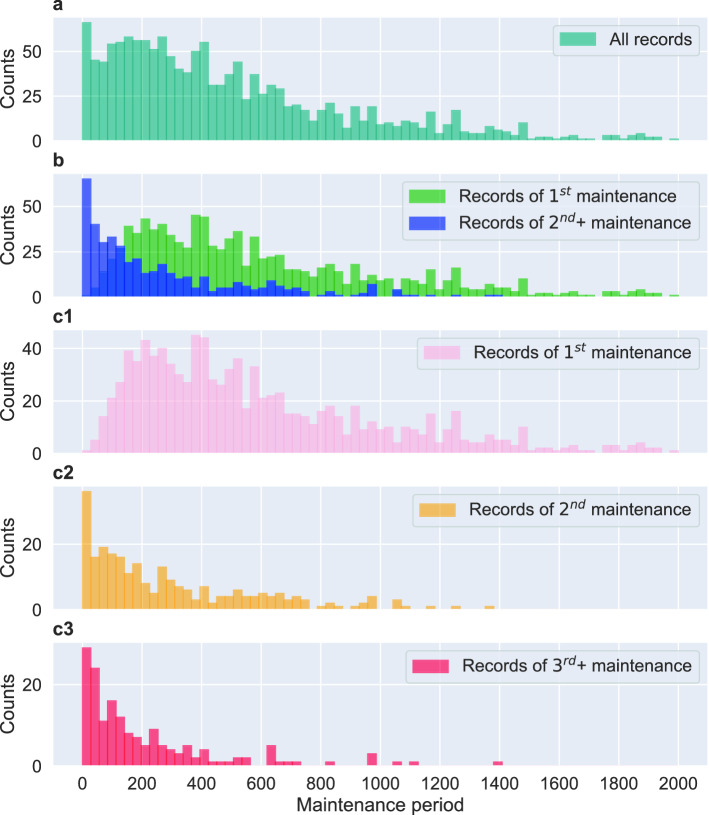
Histograms of maintenance periods for different record types. **A**: Distribution for all (uncensored) records (1, 387 records). **B**: Subdivision of the distribution in A into two types of records. The components of the $1{\rm st}$ maintenance are shown in blue (987 records) and the components of the $2{\rm nd}+$ maintenance in green (400 records.). C1–C3: A comparison between different replacement periods of records of $1{\rm st}$ maintenance (C1), $2{\rm nd}$ maintenance (C2) and $3{\rm rd}+$ maintenance (C3), corresponding to 987, 252, 148 records respectively.

During examining the distribution of the maintenance periods of all the uncensored records, see Fig. 4a, we discovered that the histogram shape of the maintenance period is right-skewed with a clear peak at the beginning. Furthermore, we noticed that there is no fade-in effect that can be observed at the start of the distribution, while there is a clear fade-out effect starting after about 400 days (see Fig. 4a). From this, we hypothesize that the distribution in Fig. 4a is a mixture of several groups (or populations) and these groups are related to the number of times a component has been maintained. Specifically, records of components that are under their $2{\rm nd}+$ maintenance can be distorted or truncated due to various reasons, e.g., distributed recordings. Interestingly, most of the maintenance periods from this group appear to locate in much earlier days, which causes such an unnatural distribution for the histogram of maintenance periods for all records. Overall, this implies that this group represents left- and interval-censored records.

In order to demonstrate this effect, we show in Fig. 4b two distributions, one for components that are under their $1{\rm st}$ maintenance and one for components that are under their $2{\rm nd}+$ maintenance. As one can see from Fig. 4b the components that are under their $2{\rm nd}+$ maintenance are prone to fail much earlier than those that are under their $1{\rm st}$ maintenance.

To further investigate this, we subdivided the events for components of $2{\rm nd}+$ maintenance in Fig. 4b (blue histogram) into records for components of $2{\rm nd}$ maintenance and components of $3{\rm rd}+$ maintenance. The distribution of their maintenance period can be seen in Fig. 4c2–c3 (c2 corresponds to components of $2{\rm nd}$ maintenance and c3 corresponds to components of $3{\rm rd}+$ maintenance). For reasons of comparison, we added also the histogram for records for components of $1{\rm st}$ maintenance. From Fig. 4c1–c3, we can see that components of $2{\rm nd}$ maintenance and components of $3{\rm rd}+$ maintenance have similar distributions. From this, we can conclude that these two groups of records are left-censored caused by multiple replacements. Furthermore, it is interesting to note that the number of records for these events corresponding to 252 and 148 which is combined much smaller than the 987 events for the 1st maintenance. Hence, there is a strong imbalance among those events.

It is important to note that when maintenance is performed for a broken component, the root cause of the breakdown remains sometimes unknown and even after changing the broken component the root cause still exists and may continue to damage the machine and its components. Therefore the second breakdown of the same component is more likely to happen much earlier than the first maintenance because there is a systemic accumulation of a hazard. In this case the actual start date of the survival records of such components that are in their $2{\rm nd}+$ maintenance period should be the date when the potential problem emerges. However it is often difficult to address the causes of a breakdown so it is impossible to estimate the actual start date of being at risk of those records.

Another issue with the components with more than one maintenance events is that the logbook for their recording is heterogeneous and subject to different operators. Specifically, the operation start date will be influenced by different operators after the machine was initiated by the manufacturer, which is potentially related to interval-censoring because the actual operation start date will somewhat deviate from the installation date. Hence, there is a heterogeneity in the documentation of the replacement dates leading to a varying quality of the records.

There is even a further issue which relates to the accumulation of hazard, mentioned above. For a conventional survival analysis, e.g., in a biomedical context, stratification is used for homogenizing groups of individuals. In our case, the multiple replacements of components and the underlying unknown cause of a failure would require such a stratification for obtaining component groups with similar properties corresponding to the same population. However, due to a lack of detailed knowledge about the underlying case, which can of course be different for different mobile work equipment, such an analysis cannot be performed faithfully.

All of these issues are difficult to address in retrospective, for this reason we filter all left-censored records in order to avoid statistical robustness issues and incorrect interpretations. Formally, this means the pre-processed data contain only uncensored and right-censored events.

#### Distributions of replacement periods for all the records from 12 individually selected components

**Figure 5:**
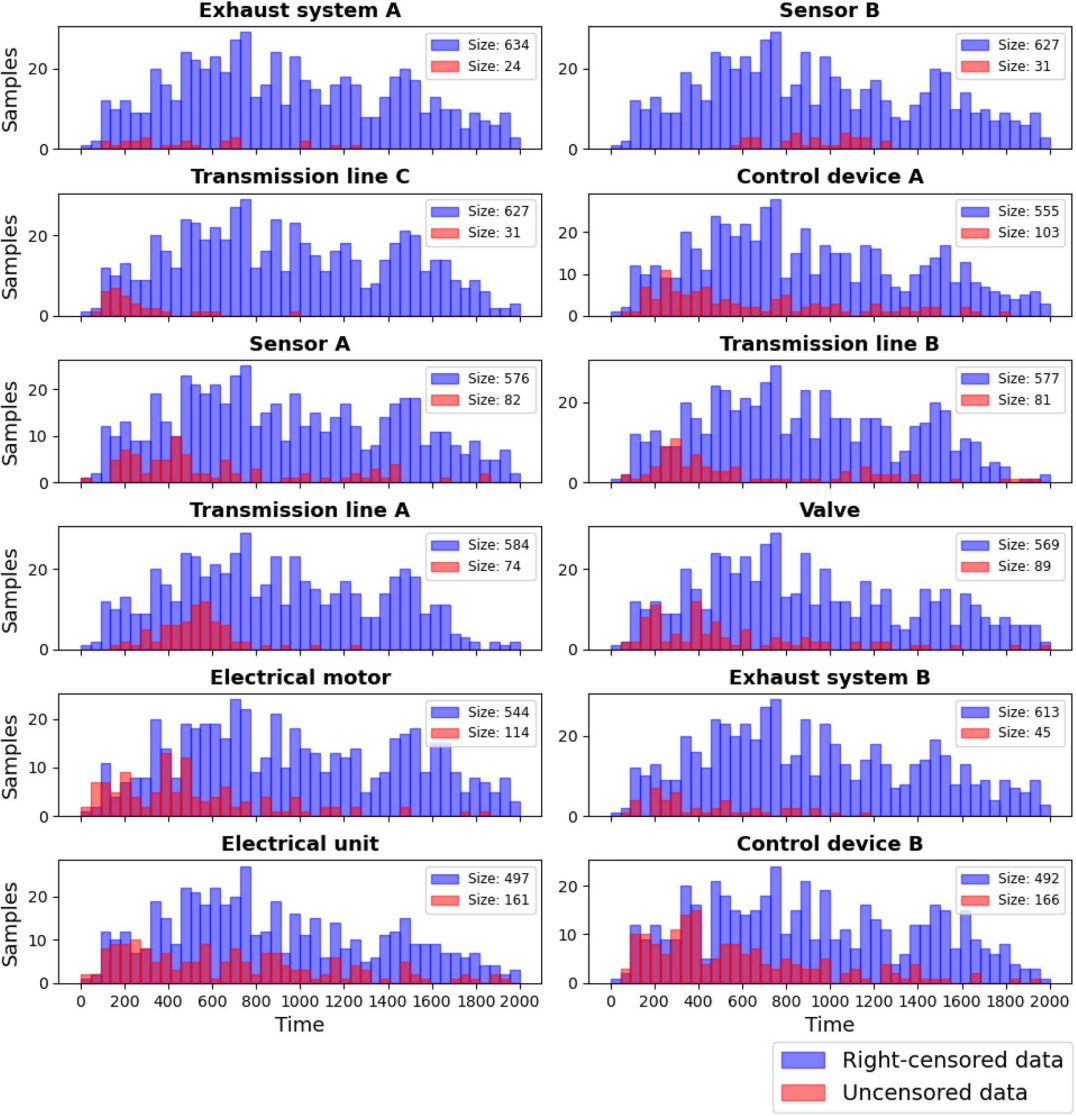
Histograms for replacement periods for all the events for 12 machine components. The right-censored events are highlighted in blue and the uncensored events are shown in red.

After the pre-processing steps, the final dataset contains in total 7,896 records consisting of uncensored and right-censored events. These events are collected from 658 unique machines thus each machine component category has 658 events (each event corresponds to a component from an unique machine), an overview of the resulting data is shown in Fig. 5. Specifically, Fig. 5 shows histograms for replacement periods from all the remaining events for 12 machine components. The right-censored events are highlighted in blue and the uncensored events are shown in red. From the Fig. 5 one can see that the right-censored events cover a wider range than the uncensored events, while most of the uncensored events are locate in early days. From the differences of the histograms, especially for the uncensored data, one can speculate about differences of the resulting hazard rates. This will be quantified in the next sections.

### Survival curves for individual components

**Figure 6:**
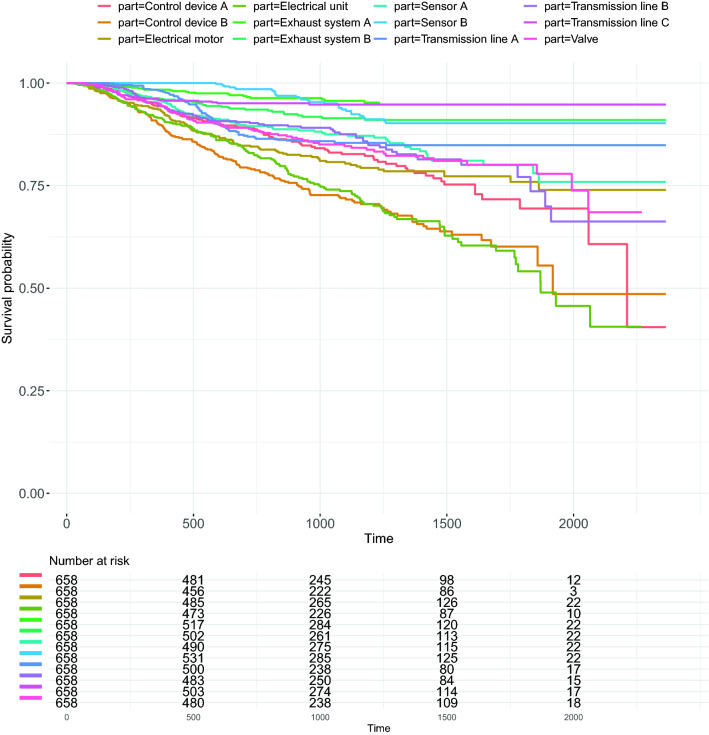
Survival curves for 12 machine components (top). The numbers at risk (bottom) give the total number of components that haven’t experienced the failure yet remaining at the different time stamps. This includes both uncensored and right-censored records.

For our first analysis for mobile work equipment components, we estimate survival curves. For this analysis, we used the Kaplan-Meier estimator, which is a non-parametric estimation technique. The resulting survival curves can be seen in Fig. 6. As one can see from Fig. 6, there can be 3 distinctive groups of survival, Electrical unit, Control device B from the high risk group, Transmission line C, Exhaust system B, Sensor A, and Transmission line A as the low risk group, and Exhaust system A, Transmission line B, Electrical motor, Control device A, Valve, and Sensor B are in the median risk group.

However, in some cases we do not have uniformly distributed failure events which cause rapid drops at the tail of the curve like Control device A, also for our data we have high percentage of censoring ranging from 74 to $96\%$ (see Fig. 5), both of these situations pose challenges for interpreting the survival probability estimated from Kaplan-Meier curve^39^. For our case, one can argue that Kaplan–Meier is inferior to other estimators such as maximum likelihood estimator or B-substitution estimator for which large sample sizes and high censoring percentage can be better dealt with^48^. However, despite of the facts that there are alternative estimators suitable for our case, one advantage for Kaplan-Meier estimator is that it does not require any prior knowledge of the distribution of survival time from the data. Therefore, it can be applied in almost all the of cases where the distributional shapes can be arbitrary, which makes the experiments much earlier when dealing with such industrial data of complex structure.

### Non-parametric and parametric estimation of hazard rates

In the following, we estimate hazard rates for the 12 mobile work equipment components. We will use parametric and non-parametric models to see if the hazard function can be described by well-defined models or if the data deviate from such parametric assumptions.

For estimating the non-parametric hazard functions we use kernel estimates implemented in the Muhaz package for R environment. The Muhaz function utilizes smoothing kernel estimators in computing the hazard rates. There are several properties affecting the resulting non-parametric hazard curve, such as the choice of the kernel, the optimizing method of bandwidth, the boundary correction type, the maximum analysis time for the estimation. In our experiments, we select Epanechnikov kernel with local optimal bandwidth as the estimator, both left and right correlation types, and the analysis time to be from 0 until the time when there are at least 10 individuals left at risks, all the parameters we choose are in align with the default parameters suggested by the coder of Muhaz^49^. For further information on how each parameter affects the resulting curve please see Section "Discussion".

**Figure 7:**
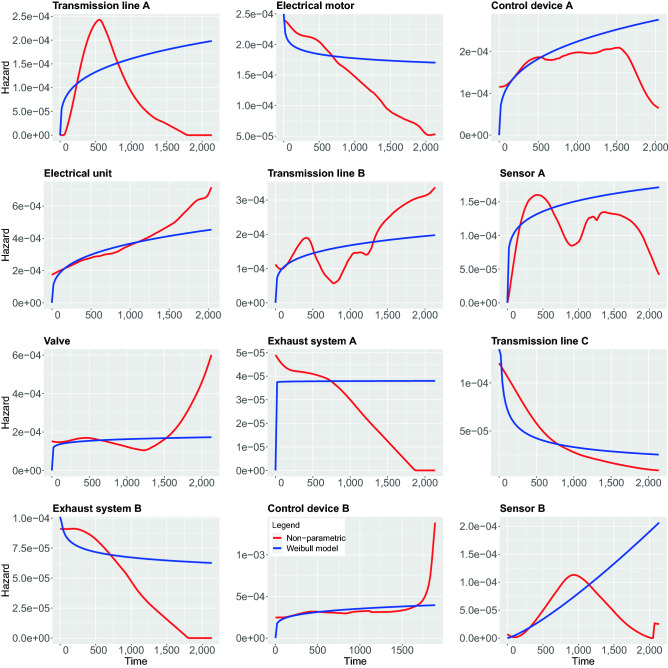
Weibull model hazard. Blue curve indicates Weibull model hazard and red curve represents non-parametric kernel-estimator hazard.

**Figure 8:**
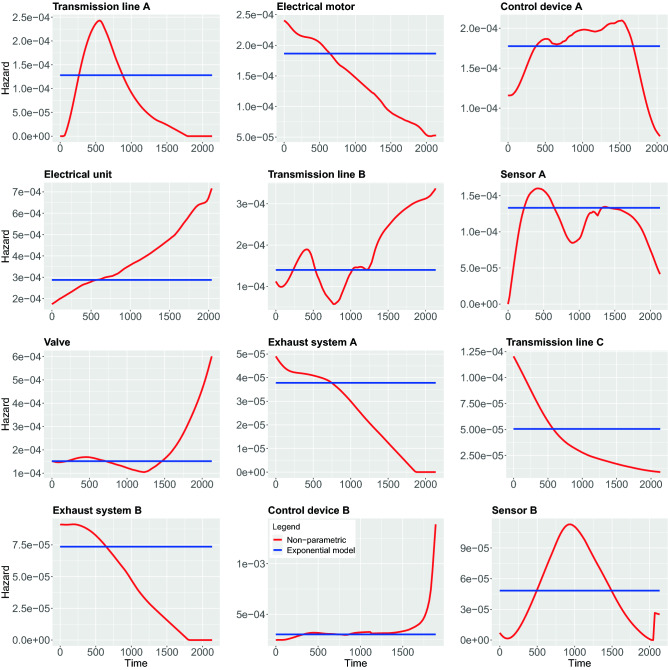
Exponential model hazard. Blue curve indicates Exponential model hazard and red curve represents non-parametric kernel-estimator hazard.

**Figure 9:**
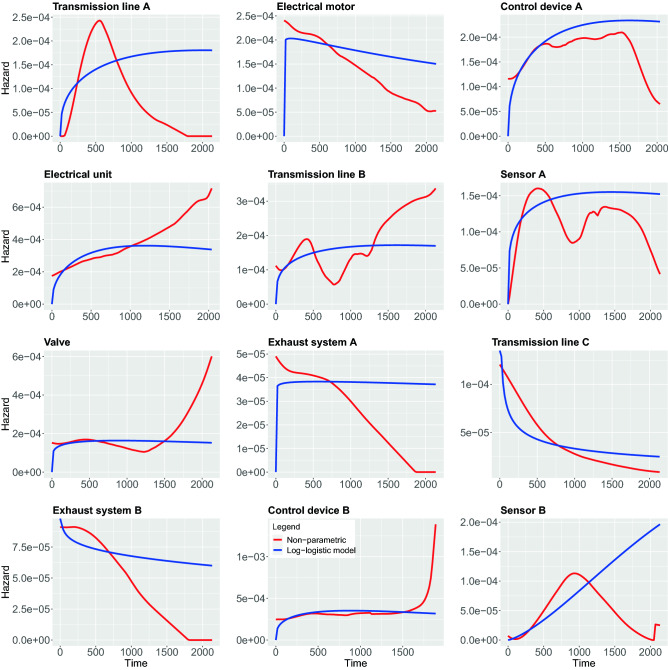
Log-logistic model hazard. Blue curve indicates Log-logistic model hazard and red curve represents non-parametric kernel-estimator hazard.

**Figure 10:**
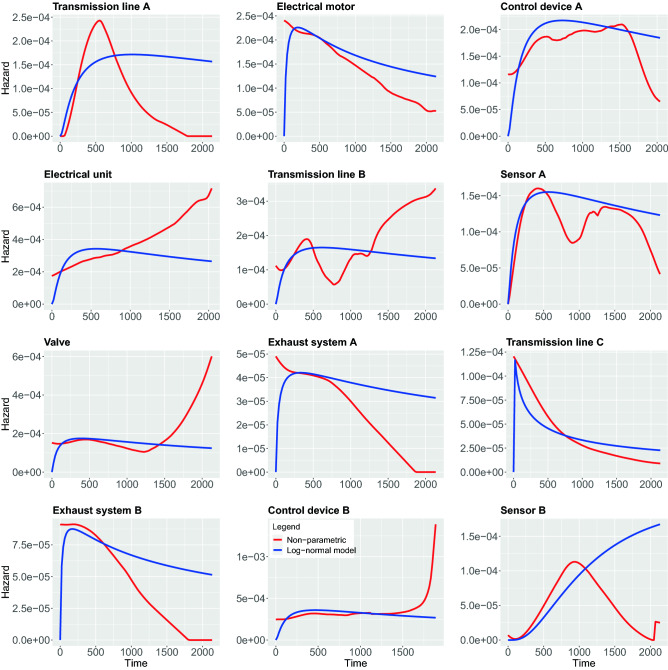
Log-normal model hazard. Blue curve indicates Log-normal model hazard and red curve represents non-parametric kernel-estimator hazard.

**Figure 11:**
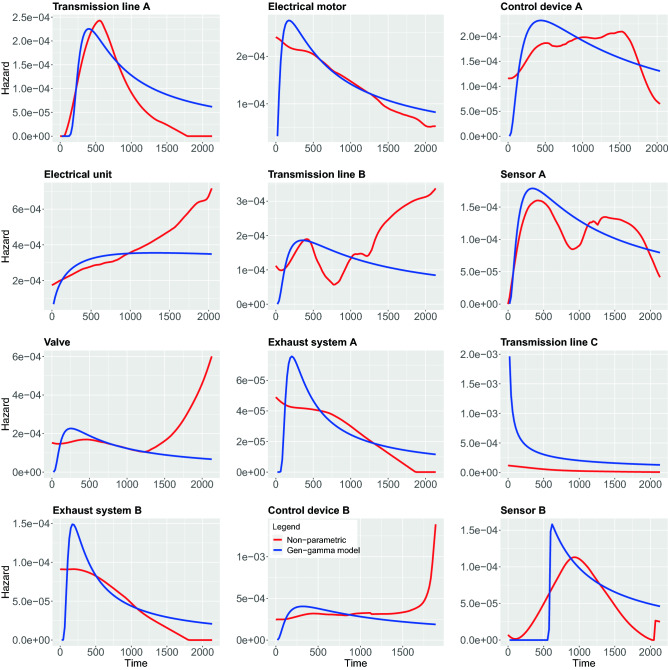
Generalized-gamma model hazard. Blue curve indicates Generalized-gamma model hazard and red curve represents non-parametric kernel-estimator hazard.

In contrast to the above non-parametric approach, using parametric models require strong assumptions about the shape of the hazard function. In the following, we study 5 different parametric models, namely, Weibull, Exponential, Log-logistic, Log-normal and Generalized-gamma. In the following, we compare both the parametric and non-parametric models by showing them in the same figure. This allows a graphical evaluation of the parametric models because the non-parametric estimations can be used as the ground truth to examine the fit of the parametric models^42^. The results of all estimations are shown in Figs. 7, 8, 9, 10, 11.

All the parametric models were fitted using flexsurv^50^ package in R environment. Flexsurv is a well wrapped function for fully-parametric modelling of survival data. It provides a collection of built-in distributions for the data and the fitting is done by maximizing the full log-likelihood.

In Fig. 7, we show results for the Weibull hazard rate. The red curves correspond to the non-parametric kernel-estimation of the hazard and the blue ones are for the parametric Weibul model (see formal definition in the Methods section). In Fig. 8, hazards with Exponential model are shown as the the blue curves while red curves correspond to the non-parametric kernel-estimation of the hazard (see formal definition of Exponential model in the Methods section). Figures 9 and 10 shows hazards with Log-logistic and Log-normal models, respectively. The red curves are the parametric hazards and the blue curves are non-parametric hazard. Figure 11 shows Generalized-gamma hazard function for 12 parts, the red curve is the Generalized-gamma hazard and blue curve is non-parametric kernel-estimation hazard. Table 1 shows the parameters estimated for each model regarding 12 parts.

**Table 1:** Estimated parameters and their standard errors for all the parametric hazard estimations from Figs. 7, 8, 9, 10, 11. Equations of the parametric models and their corresponding parameters see Fig. 1.

Parameters	Weibull		Log-normal		Log-logistic		Exponential	Generalized-gamma		
	$\gamma$	$\lambda$	$\gamma$	$\lambda$	$\gamma$	$\lambda$	$\lambda$	$\mu$	$\sigma$	$\lambda$
Transmission line A	1.275 ± 0.12	5069 ± 901	1.377 ± 0.12	8.487 ± 0.19	1.364 ± 0.13	4230 ± 717	0.0001281 ± 0.000014	6.782± 0.22	1.698 ± 0.16	−4.445 ± 0.79
Electrical motor	0.945 ± 0.07	5870 ± 1006	1.881± 0.14	8.647 ± 0.19	1.018± 0.08	4577 ± 750	0.0001864 ± 0.000017	7.983 ± 0.45	2.648± 0.25	−1.442± 0.65
Control device A	1.294± 0.10	3872 ± 497	1.410± 0.10	8.267± 0.14	1.380 ± 0.11	3277 ± 411	0.0001775 ± 0.000017	7.943± 0.39	1.911 ± 0.27	−1.079 ± 0.81
Electrical unit	1.298± 0.08	2648 ± 222	1.337± 0.08	7.781 ± 0.10	1.429 ± 0.09	2143 ± 181	0.0002875 ± 0.000022	7.854± 0.09	1.033± 0.23	0.524 ± 0.39
Transmission line B	1.215± 0.11	5101 ± 885	1.573± 0.13	8.644± 0.20	1.275 ± 0.12	4404± 744	0.0001398 ± 0.000015	8.122 ± 0.55	2.362 ± 0.31	−1.569 ± 0.98
Sensor A	1.161± 0.11	5770± 1043	1.626 ± 0.14	8.749 ± 0.20	1.224± 0.11	4903 ± 857	0.0001332 ± 0.000014	8.186± 0.48	2.444± 0.29	−1.605± 0.84
Valve	1.079± 0.09	5774 ± 1054	1.714± 0.14	8.713± 0.20	1.143 ± 0.10	4792 ± 845	0.0001516 ± 0.000016	7.600 ± 0.57	2.567 ± 0.20	−2.368± 0.93
Exhaust system A	1.003± 0.18	26186± 15960	2.285 ± 0.39	10.95 ± 0.74	1.021 ± 0.18	24074 ± 14371	0.0000378 ± 0.0000077	6.956± 1.43	4.171± 1.26	-9.684 ± 5.44
Transmission line C	0.647± 0.10	98342 ± 80848	3.339 ± 0.52	12.35 ± 0.95	0.660± 0.11	85146± 68260	0.0000505 ± 0.0000090	8.606± 0.001	0.052± 0.002	46.26 ± 6.71
Exhaust system B	0.906 ± 0.12	17633 ± 7232	2.262 ± 0.28	10.17 ± 0.47	0.935 ± 0.12	15277 ± 6073	0.0000735 ± 0.000010	6.805 ± 0.71	3.410 ± 0.53	−6.576 ± 1.97
Control device B	1.176 ± 0.07	2778 ± 256	1.390 ± 0.08	7.755 ± 0.10	1.311 ± 0.08	2151 ± 195	0.0003019 ± 0.000023	7.350 ± 0.26	1.733 ± 0.13	−1.008 ± 0.49
Sensor B	2.247 ± 0.31	4405 ± 786	0.878 ± 0.11	8.484 ± 0.20	2.339 ± 0.32	4092 ± 712	0.0000482 ± 0.0000086	6.445 ± 0.08	0.342 ± 0.22	−29.67 ± 19.7

### Assessing the parametric models of the hazard rates

In this subsection, we will access the goodness of fit of each fitted distribution by two metrics, log likelihood value (LLV), and the Akaike information criterion (AIC). The metrics are showed in the Table 2.

**Table 2:** Goodness-of-fit scores to evaluate the fit of parametric models. The studied scores are LLV (log likelihood value) and AIC (Akaike information criterion).

Metrics	Weibull		Log-normal		Log-logistic		Exponential		Gen-gamma	
	LLV	AIC	LLV	AIC	LLV	AIC	LLV	AIC	LLV	AIC
Transmission line A	−734	1,473	−726	1,456	−732	1,469	−737	1,476	** −710**	**1,427**
Electrical motor	−1,092	2,189	−1,086	2,177	−1,090	2,185	−1,092	2,187	** −1,084**	**2,174**
Control device A	−988	1,980	−984	1,973	−987	1,979	-992	1,987	**−983**	**1,973**
Electrical unit	**−1,466**	**2,937**	−1,467	2,938	−1,467	2,938	−1,473	2,949	−1,466	2,938
Transmission line B	−797	1,599	−794	1,593	−797	1,599	−799	1,601	**−793**	**1,592**
Sensor A	−812	1,629	−808	1,621	−811	1,627	−813	1,629	**−806**	**1,619**
Valve	−871	1,746	−866	1,737	−870	1,744	−871	1,745	**−863**	**1,732**
Exhaust system A	−268	540	−267	538	−268	540	−268	538	**−264**	**534**
Transmission line C	−333	671	−331	666	−333	670	−337	677	**−235**	**476**
Exhaust system B	−472	949	−469	943	−472	949	−473	948	**−463**	**932**
Control device B	−1,508	3,021	−1,500	3,005	−1,506	3,016	−1,511	3,024	**−1,498**	**3,003**
Sensor B	−327	658	−323	651	−326	657	−339	680	**−314**	**634**

Significance values are in bold.

## Discussion

Using parametric models has usually the advantage of resulting in smoother estimates of functions, in our case for hazard functions. The reason for this is that a parametric model is only capable of modeling a small number of different behaviors and, hence, averages over details in the data resulting in clean behavior of curves. Examples for such categories of behavior can be observed for the Weibull model, which allows an exponential decaying, an exponential increasing, a linear increase or a constant hazard. A more extreme example is the Exponential model, which allows only an exponential decay of hazard functions. However, in the case of an increasing hazard function the Exponential model cannot provide sensible estimates thereof.

In order to study the suitability of parametric models for estimating hazard functions, we compare them with non-parametric estimates and goodness-of-fit scores. The latter allows a quantitative evaluation. A graphical comparison of the parametric models and the non-parametric models can be found in Fig. 7, 8, 9, 10, 11 and the scores for the goodness-of-fit evaluations are given in Table 2. From a graphical comparison between parametric and non-parametric models for hazard functions, we found only a few cases providing a good qualitative correspondence. Specifically, for the Weibull and the Log-logistic model for Transmission line C and the Generalized-gamma model for Transmission line A. Furthermore, acceptable correspondence can be found for Log-logistic and Generalized-gamma model for Sensor A and for the Weibull model for Electrical unit. Interestingly, the Exponential model is the worst because its constant behaviours for the hazard function provides the least flexibility which is not appropriate for any components studied in this paper. From the AIC and LLV values in Table 2 one can see that the Generalized-gamma model basically outscores every other parametric model for all components. Specifically, for the Transmission line C the AIC score for the Generalized-gamma model is almost half of other parametric models’, and for Sensor B the AIC score is 10% less than for the others’ AIC scores. This confirms the power of the Generalized-gamma model and its flexibility. All scores from the other parametric models, i.e., for Weibull, Log-normal, Log-logistic and Exponential model are pretty close to each other and the differences are almost within 1%. Overall, as one can see, the monotonic hazard behaviour is rather easy to be modeled by a parametric model, and a gamma-shape hazard can be captured by a Generalized-gamma model. However, most of the hazard rates with sophisticated, complex pattern can not be captured by any parametric model. Hence, in general, parametric models for the hazard are not capable of providing very precise estimates of the true underlying distribution. That means, except for the few case discussed above, the hazard functions of the components are best fitted using non-parametric models.

From the estimates of the non-parametric models, we can distinguish between a number of different main behavior-types of the hazard functions. These can be summarized as follows:

- Decaying
- Gamma-shaped
- Oscillating
- Increasing

For the oscillating behavior, it is clear that none of the parametric models can fit this behavior because none of the parametric models is capable of exhibitting such a complex behavior. This means also that Transmission line B and Sensor A provide the most complex hazard functions.

For the Gamma-shaped behavior one can find parametric models for Transmission line A, as discussed above. However, the behavior of Sensor B and Control device A is more Bell-shaped (i.e. symmetric) and for this reason also this behavior is outside the capabilities of the parametric models.

For the remaining two behavior-types of the hazard function, namely, for decaying and increasing hazards, their behavior is more subtle. Specifically, while Valve, Electrical unit and Control device B are increasing one can observe two subcategories thereof. The first one starts from a plateau and increases steeply toward the end, while the second one shows a linear increase. Again, none of these behaviors is part of the repertoire of parametric models. Finally, also for the decaying behavior one observes two subcategories. The first shows a linear decaying (Electrical motor, Exhaust system A and Exhaust system B) while the second one is exponential.

Overall, from our discussion above follows that there are four main behavior-types of the hazard functions. However, in addition there are further subcategories that are crucial to recognize because some allow the usage of parametric models while others do not.

Non-parametric hazard estimator is capable of producing any arbitrary hazard shapes thus complicated hazard behaviours can be captured and described from the non-parametric estimation and non-parametric hazard can be used as the ground truth to validate the parametric hazard estimation. However, kernel-smoothing based non-parametric estimations of hazard is unstable and sensitive to parameters such as the minimal and maximum analysis time, kernel types, bandwidth and correlation type, in addition, different components generally require different combinations of the parameters in order to get be best estimation, and this is the reason why for some cases we see that the parametric estimation does not match the ”ground truth” provided by the non-parametric estimation even though both of them were estimated from the same data. In our study, we fine-tuned the parameters and found that the choice of the kernel has only a minor influence on the smoothness of the estimated curve. For our data the Epanechnikov kernel seems to provide the most smooth estimation near the boundary area. The range of analysis scope has large effect on the estimation near the boundaries of the curve and even further influences the overall shape of the curve, one needs to be careful when choosing the time interval of the analysis scope since the lesser events left the more biased the estimation will be. The bandwidth influences the smoothness of the curve and choosing the optimal bandwidth is a trade-off between the variance and bias of the estimated curve. Different types of boundary correlations reshape the curve near the left or right boundaries. Applying left or left-right boundary correlations gives most of the time a smoother estimation near the boundaries but sometimes the resulting curve has a completely opposite hazard behaviour compared to the curve without any boundary correlations. Hence, one needs to carefully examine the data in order to select the right approach. In conclusion, we found that our choice of parameters corresponds to the parameters suggested by the Muhaz package^41^ and their robustness is confirmed by the application in several other studies.

As discussed in Section “Data pre-processing”, our underlying raw data are subject to various types censoring including left-, left-interval, and right censoring. Due to the complicity of the censoring types we could not utilized the left- and left-interval censored records, however, we found that after removing the left- and interval- censored data the histogram of replacement period for uncensored and right-censored data still somewhat represent two different populations within each component in Fig. 5, which might imply that the estimations could be further improved.

One challenge we recognized is that the maintenance periods could use the cumulative working hour instead of taking the differences of initial day and the maintenance date. For our study, we assumed that all machines are operated according to similar schedules in order to calculate the maintenance date by taking the difference between the installation date and the maintenance date. However this may not always be the case when machines are from different operators and subject to different workloads and/or work conditions.

Furthermore, the records we collected were categorized for 12 different components, hence the categories of records do not consider some potential risk factors that are very much likely to affect the survival time on individuals differently within each category. Those factors might be different types of usage, different driver working styles, how heavily the machine was working before the breakdown of a certain part, or some external environment conditions like weather, humidity and anything that might affect the condition of individual machines. A reflection of those unidentified factors may be visible in Fig. 7 to 11 causing the oscillating in the non-parametric estimation of hazards for the component Transmission line B and Sensor A. In order to identify more sub-groups within each component more detailed recording is needed.

In the introduction, we discussed publications utilizing survival analysis for studying predictive maintenance or related problems. However, those studies are different to our study with respect to the following points. First, some studies use only parametric models, e.g.,^22^. Second, the studies that use non-parametric models have a different focus. Specifically, in^28^ data for an Air Force system were simulated to test three different estimators of survival functions. That means neither real data have been used nor hazards were studied. While in^24^ non-parametric estimates for Kaplan-Meier curves and hazard rates were used the resulting hazard functions show only a simple non-linear increasing that should be captured by a parametric Weibull model. Hence, no complex pattern of hazard rates were present nor were different types of censoring discussed. Third, most studies focus on making predictions on the remaining life, utilizing in addition to time-to-event data information about covariates, e.g., sensor information. In contrast, we do not utilizes such covariates in our study but use only time-to-event data for modeling failure probabilities. Forth, the pre-processing of the data, including the identification and cleaning of censoring-related issues is usually completely omitted in other papers. Instead, in our paper one of our main focus is on the pre-processing, which is also a key in getting accurate estimates from data and in preventing analysis errors. We hope that this important topic will be recognized by others because we think that the presence of different types of populations, giving rise to different types of censoring, is not unique to our study by a general problem but it has been overlooked so far.

Finally, we would like to note that in recent years, there is increasing interest in combining machine learning and survival analysis^29,30^. However, in our opinion and based on the results obtained in our study, we think that many of such studies are premature. Specifically, we demonstrated the importance of identifying different types of censoring and the validity of parametric of non-parametric models to obtaining an understanding of prognostic modeling of machine components for a particular data set underder investigation. Only after such a thorough analysis extensions can be addressed.

## Conclusion

In this paper, we presented a survival analysis framework for prognostic maintenance employing parametric and non-parametric estimators for survival probabilities and hazard functions of machine components from mobile work equipment.

From an exploratory analysis of the raw data of maintenance records, we found that these records contain a mixture of left-, interval- and right-censoring events. This is further complicated by the imbalanced presence of these censoring types, a heterogeneous quality of recordings for multiple replacements and a diversification of the machine population. In order to improve the robustness of statistical estimators used for estimating survival curves and hazard rates and for simplifying the analysis and its interpretations, we filtered the data leaving only right-censored records. This standardizes the data and makes it amenable for well-developed survival analysis methodology.

For the analysis of the pre-processed data, we utilized Kaplan-Meier estimators for the survival probability of 12 machine components. From this, we noticed various groups hinting at different survivability characteristics. This impression could be further quantified by estimating the corresponding hazard rates. Using 5 different parametric and non-parametric kernel estimation we found not only complex patterns, including decaying, oscillating and increasing behavior, but also subgroups thereof, e.g., with respect to a linear or exponential decaying. Hence, the observed impression about the survival curves manifested in quantitatively distinct shape-types of hazard rates. Furthermore, we accessed the goodness-of-fit of the parametric models both graphically and quantitatively. From this, we found most machine components are best modeled using non-parametric estimators, however, notable exceptions are Transmission line A and Transmission line C which can be described by a Generalized-gamma respectively Weibull model.

Overall, our analysis revealed that the application of a survival analysis framework is involved requiring already an advanced data pre-processing to adequately identify different types of censoring records before the actual analysis. Also the observed shape of hazard rates is very rich and complex indicating considerable challenges for the operational management of predictive maintenance, e.g., for organizing efficient supply chains. Furthermore, the knowledge we gained from our survival analysis works could support our future application as we have seen from the publications where survival analysis results can be further incorporated into actual predictive maintenance applications.
